# Considering the Specific Impact of Harsh Conditions and Oil Weathering on Diversity, Adaptation, and Activity of Hydrocarbon-Degrading Bacteria in Strategies of Bioremediation of Harsh Oily-Polluted Soils

**DOI:** 10.1155/2017/8649350

**Published:** 2017-01-24

**Authors:** Zulfa Al Disi, Samir Jaoua, Dhabia Al-Thani, Saeed Al-Meer, Nabil Zouari

**Affiliations:** ^1^Department of Biological and Environmental Sciences, College of Arts and Sciences, Qatar University, P.O. Box 2713, Doha, Qatar; ^2^Central Laboratories Unit, Qatar University, P.O. Box 2713, Doha, Qatar

## Abstract

Weathering processes change properties and composition of spilled oil, representing the main reason of failure of bioaugmentation strategies. Our purpose was to investigate the metabolic adaptation of hydrocarbon-degrading bacteria at harsh conditions to be considered to overcome the limitations of bioaugmentation strategies at harsh conditions. Polluted soils, exposed for prolonged periods to weathered oil in harsh soils and weather conditions, were used. Two types of enrichment cultures were employed using 5% and 10% oil or diesel as sole carbon sources with varying the mineral nitrogen sources and C/N ratios. The most effective isolates were obtained based on growth, tolerance to toxicity, and removal efficiency of diesel hydrocarbons. Activities of the newly isolated bacteria, in relation to the microenvironment from where they were isoalted and their interaction with the weathered oil, showed individual specific ability to adapt when exposed to such factors, to acquire metabolic potentialities. Among 39 isolates, ten identified ones by 16S rDNA genes similarities, including special two* Pseudomonas* isolates and one* Citrobacter *isolate, showed particularity of shifting hydrocarbon-degrading ability from short chain* n*-alkanes (*n*-C12–*n*-C16) to longer chain* n*-alkanes (*n*-C21–*n*-C25) and vice versa by alternating nitrogen source compositions and C/N ratios. This is shown for the first time.

## 1. Introduction

Remediation of contaminated sites with oil components is a major concern, especially because the spread of dangerous molecules in the environment and their transfer into the groundwater and the food chain [1]. On the other hand, exorbitant costs are incurred by rehabilitation projects which require soil excavation and expensive clean land transport to remediation units [2]. In practice, the techniques based on heat and physicochemical treatment are most common, while biological processes are often considered reserved for a narrow category of compounds as light petroleum hydrocarbons or adapted to specific conditions [3].

Biological techniques that use the power of decontaminating microorganisms that are grown on contaminated soils are known and used for many years. Some are implemented since the 80s and 90s and came to maturity; others are the subject of research and development [1]. The interest of these technologies lies in the fact that they require no excavation or transport, with exceptions, which makes their implementation much cheaper.

These are microorganisms that are used as biological systems. The phenomenon of biodegradation can occur spontaneously, as natural attenuation [4] in the presence of air or absence of oxygen. Microorganisms grow and/or obtain energy by use of molecules of pollutants. In this case, called cometabolism, the microorganism derives no direct benefit. Complete biodegradation implies detoxification of pollutants to the stage of dioxide carbon, water, and harmless mineral salts [5, 6]. But, incomplete biodegradation can provide products of degradation which are less toxic than the original pollutant, but not necessarily. For example, biodegradation of trichloroethylene or tetrachloroethylen can release vinyl chloride which is more toxic and carcinogenic than the parent compound [7–9]. Indeed, very often, natural conditions are not favorable enough, for example, with lack of nutrients, pH, redox potential, oxygen, or suitable bacteria [10]. In such situations, the process can be improved by optimizing the necessary factors. For example, in 1989, during the oil spill Exxon Valdez due to the supertanker accident (spilled 201,000 m^3^) to accelerate the degradation of oil hydrocarbons, needed nutrients were spread on 1000 miles along the coast of Alaska [3].

In the field of bioremediation, it will be more likely to observe the speed of the natural biodegradation and intervene only if the natural activity is not sufficient to eliminate the pollutant, fairly quickly [11]. The intervention is more likely necessary with intensive oil weathering processes that effect biodegradation. These processes change properties and composition of spilled oil. On the other side, biodegradation is influenced by abiotic factors such as pH, redox potential, temperature, moisture, oxygen, nutrient availability, and soil salinity. Composition of the mixture of hydrocarbons determines their solubility and resistance to degradation. Similarly, the interaction of pollutants with soil components determines the effectiveness of bioremediation [12]. In soils exposed over long periods, hydrocarbons are strongly adsorbed to particles and/or incorporated in very fine pores, making them inaccessible to microorganisms [13]. Bioavailability of hydrocarbons is a key factor that may often limit the effectiveness of bioremediation processes [14–16] and especially bacterial consortia. Under specific conditions, a process of biopolymerization of compounds via a process of bioautoxidation, producing intermediate degradation products, might occur, resulting in the production of nonbiodegradable polymers.

Bacteria, through metabolic pathways, take the central role of the phenomena of biodegradation of hydrocarbons. Some of them have developed a system of signaling, chemotaxis, facilitating access to hydrocarbons [14–16]. These mechanisms operate via chemoreceptors and signaling pathways and cause a displacement of the bacteria according to the gradient of concentration of the pollutant. Bacteria then accumulate at the interface between the hydrophobic pollutant and the hydrophilic medium, causing an increase in the rate of degradation of compounds and often an increase in their desorption [17]. Again, the weathered spilled hydrocarbons would limit the interaction of bacteria and pollutants. Somehow, the microbial communities are able to degrade pollutants under the influence of environmental parameters, considering properties of pollutants and microflora in the soil [18]. Microorganisms of a soil never having been exposed to contaminating compounds do not have necessarily the ability to metabolize pollutants. However, when exposed to such compounds, they are often able to adapt [19, 20]. A set of biotic and abiotic factors in each polluted area should guide and orient the biodegradability of the corresponding pollutants [21]. This represents the main origin of failure of most of the bioremediation applications based on bioaugmentation for cleaning up polluted areas with hydrocarbons. Many areas, including the gulf area, including Qatar, are characterized with special soil, water, and weather conditions affecting the chemical structures of oil components and microbial community. The extreme weather in Qatar leads to prolonged periods of adapted microorganisms' selection [16]. These stress conditions may provide very special and interesting biological degradation routes, making expectations of local microbial isolates exhibit originality and novelty. Harsh soils from this region could be a source of interesting information for development of appropriate bioremediation approaches [21].

C/N/P ratios are a general bioremediation practice. C/N/P ratios supporting good hydrocarbons degradation are lower than the general bacterial requirement due to the low bioavailability of hydrocarbons and a different elemental cell composition of the produced biomass. For most pollutant compounds, the carbon is mostly not substantially incorporated into microbial biomass, but a large fraction is mineralized to CO_2_ and H_2_O for the production of energy. It has been reported that growth on PAHs of single strain* Pseudomonas* and* Rhodococcus* cultures is characterized by relatively high mineralization yields at specific C/N/P ratios affecting the mineralization. Also, the cell production and accumulation of metabolites are largely dependent on the C/N/P ratios [22].

In this work, we intend to isolate, screen, and select bacterial strains from harsh Qatari soils, taking into consideration weather and weathering processes, as previously decribed [23]. By understanding how the extreme weather in Qatar leads to prolonged adaptation periods, best bacterial candidates that could be selected for implementation of appropriate bioremediation strategies at similar conditions worldwide could be selected. This represents a key parameter in selecting appropriate isolates for bioremediation of a specific oily-polluted site, avoiding frequent failure of bioaugmentation strategies at harsh conditions.

## 2. Materials and Methods

### 2.1. Strategy for Enrichment, Isolation, Identification, and Activity Study of Hydrocarbon-Degrading Bacteria

**Figure 1:**
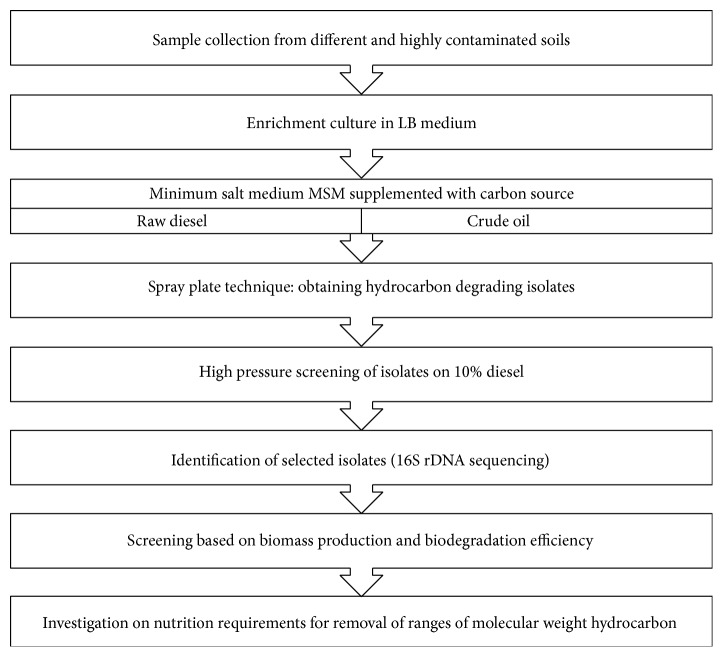
Isolation strategy for isolation and screening of the hydrocarbon-degrading bacteria under high selection pressure.

The strategy followed in our work to isolate hydrocarbon-degrading bacteria with high growth and biological activity is described in Figure 1. The related materials and methods were adapted accordingly.

### 2.2. Soil Samples

Seven samples were collected from various oil-contaminated sources. The locations were selected to ensure that the oil components were exposed for long time (exceeding 5 years) to extreme weather and thus weathered, and indigenous bacteria adapted. Autoworkshops were judged very useful, especially in the sites contaminated with lubricants, diesel, and gasoil leaks as well as in the repairing area. The other samples were from 3 different oil-industry waste management sites in Qatar, where spilled oil and exploitation byproducts are being stored and highly controlled (Dukhan sites).

Sampling was performed using a sterile spatula at a tillage depth of 1-2 cm, randomly from different points. The soil samples were collected and placed into sterilized glass bottles, properly sealed, labeled, and warped with foil to prevent any further light reactions. All collected samples were temporally stored in an icebox at 4°C and then transferred to the laboratory for further analysis. The pH of the collected soils ranged from 7.0 to 7.5, while the temperatures ranged from 35 to 40°C at the sampling times. The annual average of temperature in Qatar is around 30 ± 2°C (Ministry of Baladya and Environment Qatar, personal communication), which was selected to perform all the experiments.

### 2.3. Culture Media

Luria Broth (LB) medium was used for the first enrichment cultures in the program and the purification of isolates. Further enrichments and biodegradation experiments were performed in liquid Minimum Salt Medium (MSM) with a pH of 7.2, selected as an average pH of the used soils samples and containing the following per liter (with a pH of 7.2): a nitrogen source (either ammonium nitrate (NH_4_NO_3_), ammonium chloride (NH_4_Cl), or sodium nitrate (NaNO_3_) as specified in each experiment), 4.0 g; Na_2_HPO_4_, 2.0 g; KH_2_PO_4_, 0.53 g; K_2_SO_4_, 0.17 g; MgSO_4_·7H_2_O, 0.10 g, and 1 mL of trace element solution composed of (per 100 mL) EDTA, 0.1 g; ZnSO_4_, 0.042; MnSO_4_, 0.178 g; H_3_BO_3_, 0.05; NiCl_2_, 0.1 g. All media were sterilized at 121°C for 20 min. Solid MSM contained agar 15 g/L. Diesel as sole carbon source was then added at 10% (v/v) in a final culture volume of 20 mL. Diesel stock was kindly provided by Um-Said refinery (personal communication), with a complete analysis, showing hydrocarbons composition ranging from C-12 to C-25. It contained 750 g/L carbon. For calculation of the C/N ratios, the sole carbon source was diesel, and the sole nitrogen source was without carbon content, either ammonium nitrate (4 g of NH_4_NO_3_ containing nitrogen 1.4 g), ammonium chloride (4 g of NH_4_Cl, containing nitrogen 1.05 g), or sodium nitrate (4 g of NaNO_3_, containing nitrogen 0.66 g).

### 2.4. Isolation of Hydrocarbon-Degrading Bacteria

A total of 2.5 g from each sample was suspended in 25 mL of LB as the first enrichment medium in order to promote spore's germination and bacterial growth with less stress than that with oil or diesel and shortening the lag phase of bacteria growth as well. This step was necessary to isolate most of hydrocarbon-degrading bacteria from each soil, since, in a preliminary study (not shown), a maximum of 3 isolates was obtained from each soil. The liquid cultures were incubated at 30°C in a rotating shaker set at 300 rpm for 3 days. Then, 2 mL from each liquid culture was transferred to 25 mL of the second enrichment medium which was MSM liquid medium supplemented with 1 mL of crude oil (or diesel) as the sole carbon source. This step of the adaptation of the microorganisms to oil/diesel as the sole carbon source was repeated twice to enrich the media with microorganisms only able to grow using oil and diesel components as the carbon source. The spray plate technique [24] was also used to spread 100 *μ*L of the second MSM enrichment cultures (liquid MSM cultures) on the MSM agar medium, and then 100 *μ*L crude oil/diesel was spread on the surface of the MSM agar. The plates were wrapped with aluminum foil and incubated in the dark at 30°C for 2 weeks. Colonies forming clear zones on the coated solid MSM medium were selected. The isolates were purified using subculturing by streaking on LB-agar plates. Their ability to grow and form white colonies on crude oil-coated MSM plates is then checked before preservation in 30% glycerol at −80°C.

### 2.5. Screening of Diesel-Degrading Isolates Based on Biomass Production

The selected isolates, considered candidates for the degradation of petroleum components, were first cultured into 10 mL of LB broth for 48 h at 30°C. Then, the optical density (OD) of each culture was determined at 600 nm. Each isolate was used to inoculate a sterilized 20 mL MSM supplemented with diesel 10% (v/v), so that the initial OD was of 0.15 and incubated during 2 weeks at 30°C. The growth was then evaluated by spreading 100 *μ*L of serial dilutions in the MSM medium on LB plates. Colony forming units (CFU) were used after enumeration as cell counts in each culture broth.

Experiments were carried out in triplicate and included two controls. One culture control was performed with noninoculated MSM-diesel and used for analysis of diesel hydrocarbons incubated at the same conditions as the inoculated MSM-diesel cultures. The second culture control was performed with inoculated MSM medium with each isolate, but without diesel, to show if the isolates can grow using EDTA. All the isolates were shown to not grow at all without diesel as shown by CFU which was at the most equal to the initial cell counts of the inoculated cultures at the beginning of incubation.

### 2.6. Molecular Identification of Isolates

DNA was obtained from cells after overnight growth in LB plates. The cells were suspended in 0.5 mL of distilled water, boiled for 10 min in a water bath, and then centrifuged for 10 min at 13,000 rpm. The supernatant (total DNA) was placed in a new tube for PCR amplification.

The 16S rDNA fragment (1500 bp) was amplified using universal primers: RibS73sp 5′-AGAGTTTGATCCTGGCTCAG-3′ and RibS74sp 5′-AAGGAGGTGATCCAGCCGCA-3′.

The sequencing of bacterial 16S rDNA amplicons was performed after purification, using an Applied Biosystems 3500 Series Genetic Analyzer System. The obtained 16S rDNA sequence of each isolate was used to determine the most closely related sequence of available sequences in the Gene Bank database using the Blast server at NCBI.

### 2.7. Analysis of Diesel Degradation by Gas Chromatography (GC)

GC analysis was performed after the incubation periods using a Perkin Elmer Clarus 680 GC, FID detector at a 150°C injector temperature using Column Elite-1 (Dimensions: *L* 60 m, ID 0.25 mm, and DF 0.25 *μ*m). The diesel layer was carefully extracted using a micropipette, placed into a sterilized Eppendorf tube, and centrifuged for 1 min at 13,000 rpm to separate any remaining liquid medium, and then the pure diesel layer was transferred to a new sterilized Eppendorf tube and used for GC analysis. The removal of diesel components was determined by the reduction in the area under the hydrocarbon peaks in the chromatograms when compared to that of the abiotic control, suggesting the removal of diesel components. The raw diesel, obtained from Um-Saeed Refinery (Qatar), is composed of hydrocarbons containing 12 to 25 carbon atoms (personal communication).

The removal of diesel hydrocarbons as a whole was expressed as the percentage of removal in relation to the amount of the remaining fractions in the appropriate abiotic control samples.

The removal efficiency (RE), based on the reduction in the peak area of selected hydrocarbons from the chromatogram of diesel from the control culture, was evaluated using the following expression: RE (%) = 100 − (*A*_s_ × 100/*A*_c_), where As is the total area of the peak in each sample, Ac is the total area of the peak in the control, and RE (%) is the efficiency of removal [25].

The solubilized hydrocarbons in the culture broth (aqueous phase) were at negligible concentrations as measured by a published method of Mnif et al. [26]. 4 mL of the centrifuged culture broth (aqueous phase only) of each isolate, at 10,000 rpm for 10 min, was mixed with equal amount of hexane for extraction of solubilized hydrocarbons. After a vigorous vortexing for 3 min followed by centrifugation (4,500 rpm for 15 min), the optical density in the hexane phase was measured spectrophotometrically at 295 nm. The concentration of hydrocarbons extracted by hexane was calculated from a calibration curve performed with different concentrations of diesel in hexane ranging from 0 to 100 *μ*g/mL hydrocarbons. The concentrations were often ranging from 2 ± 2 to 8 ± 3 *μ*g/mL hydrocarbons, knowing that diesel represented 10% (75 mg/mL) at the beginning of the isolates culturing.

### 2.8. Statistical Analysis

Three replicates were used throughout the experiments, and the mean values with standard deviations were calculated using Microsoft Excel 2016. The significance of removal, shifts, and variations in removal efficiencies was analyzed using ANOVA at 95% confidence level.

## 3. Results

### 3.1. Isolation of Hydrocarbon-Degrading Bacteria from Soils Polluted with Weathered Hydrocarbons

**Figure 2:**
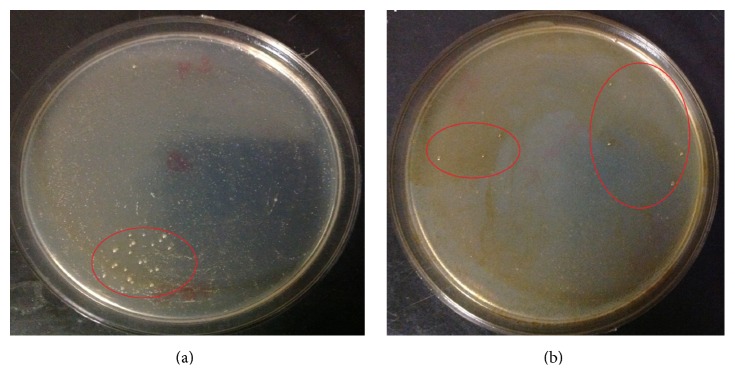
Sample of oil-coated and diesel-coated plates with one isolated strain HDB8. (a) Diesel-coated plate; (b) oil-coated plate.

In order to investigate their adaptation consequences, hydrocarbon-degrading bacteria were isolated following a specific strategy focusing on adapted bacteria to extreme weathering processes of hydrocarbons, as well as drastic growth conditions. The enrichment steps in MSM-10% of diesel or crude oil represented a high selecting pressure targeting isolation of highly tolerant hydrocarbon-degrading bacteria.  Figure 2 shows a sample of coated plates with growing colonies. Following this program, 39 bacterial isolates were separated as pure cultures, from the seven samples and using the two carbon sources (diesel or crude oil) in enrichment cultures.  Table 1 shows the distribution of the isolates. Many of the isolates were able to grow on oil-coated MSM plates, but not on diesel-coated ones, even though being issued from the same sample. Other isolates showed the opposite. Bacteria isolated from the sample of diesel leaking site of the autoworkshop 2 as well as those of the oil waste management site 2 were not able to grow on oil-coated MSM plates. Bacteria isolated from the repairing area of the autoshop 4 were not able to grow on diesel-coated MSM plates. From the other samples, it is not excluded that some of the isolates were growing on both media. The number of isolated bacteria growing on oil or diesel plates differed from one sample to another. The highly selective isolation program employed in our study allowed selecting isolates, potentially exhibiting various metabolic characteristics, and tolerance to hydrocarbons toxicity in agar plates coated with diesel or crude oil. The behavior of each of them in liquid media may change, needing further investigations.

### 3.2. Screening of Bacterial Isolates Based on Growth in 10% Diesel

Growth and tolerance to toxicity of the isolates were evaluated in liquid MSM with 10% diesel as the sole carbon source which may lead to screening the bacterial isolates based on their ability to grow at such toxic concentration. The criterion of selection was the cells' biomass (cell counts) after 2 weeks of incubation. The cell counts were considered as indicator of the use of diesel hydrocarbons (sole carbon and energy source) at the experimental conditions. In addition, the analysis of the residual hydrocarbons in the medium after the incubation period was used to evaluate the performance of the isolate to degrade the diesel components.  Table 2 shows the results obtained with the best 10 isolates which produced more than 10^7^ cfu/mL. With isolates providing lower final cell counts, hydrocarbon removal efficiency was not significant as calculated from the GC analysis (not shown). These isolates as well as those listed within the 10 isolates (Table 2) but showing removal efficiencies lower than 5% may degrade small fractions of some diesel components or may not be able to degrade hydrocarbons; they may just convert some to intermediates. Three isolates (HDB8, HDB9, and HDB38) showed hydrocarbons removal efficiencies above 19% with cell production of 26 × 10^7^ cfu/mL to 33 × 10^7^ cfu/mL. The isolate HDB18 was characterized by a high growth of 20 × 10^7^ cfu/mL, but corresponding to a relatively low hydrocarbons removal of 10%. Considering the yield “cfu/removed hydrocarbons,” these isolates showed significant differences. Indeed, the initial culture medium contained 75 mg/mL carbon from 10% diesel. By calculating the removed quantity by each isolate and the corresponding cfu produced on such removed quantity, the yields were 1.97, 1.82, 1.42, and 2.67 × 10^7^ cfu/mg of removed carbons with HDB8, HDB9, HDB38, and HDB18, respectively. By combining these biomass production yields to the final cell counts and removal efficiencies for the 4 isolates, it is obviously clear that each isolate was exhibiting a different metabolism to convert certain diesel hydrocarbons to new cells, or other end products, including intermediates. Despite its low overall removal efficiency, HDB18 has the highest potentialities to extract energy from the total or partial oxidation of hydrocarbons and use it for growth. However, in such a complex medium containing lots of diesel substrates, it is necessary to consider the potentialities of each isolate to attack ranges of diesel hydrocarbons. But, the merit of this study was to show variations in the metabolism of the hydrocarbon-degrading bacteria in contact with a mixture of substrates.

### 3.3. Identification of the Selected Isolates

The 10 preselected isolates, showing high growth (above 10^7^ cfu/mL) in 10% diesel, were identified based on the sequencing of bacterial 16S rDNA amplicons after purification.  Table 3 lists the identified isolates HDB8 and HDB9 and HDB38 which were* Pseudomonas aeruginosa*, which is the most reported genus in hydrocarbon degradation [1, 27, 28]. HDB18 was* Citrobacter amalonaticus* which was also reported [29, 30]. By combining the biological activity and the identity of each isolate, it may be concluded that even though belonging to the same genus and species, HDB8 and HDB9 exhibited different metabolism potentialities when considering the yields of growth by consuming diesel hydrocarbons. It is to be noticed that HDB8 and HDB9 were isolated from the same soil, sampled from autoworkshop 3 (oil waste tanks). They might develop different ways of adaptation, representing an interesting tool to investigate adaptation consequences. These two* Pseudomonas aeruginosa* isolates (HDB8 and HDB9) as well as HDB18* (Citrobacter amalonaticus)* were selected for further investigations of their adapted metabolisms.

### 3.4. Metabolism Potentialities of Selected Isolates, for Diesel Hydrocarbons Degradation

**Figure 3:**
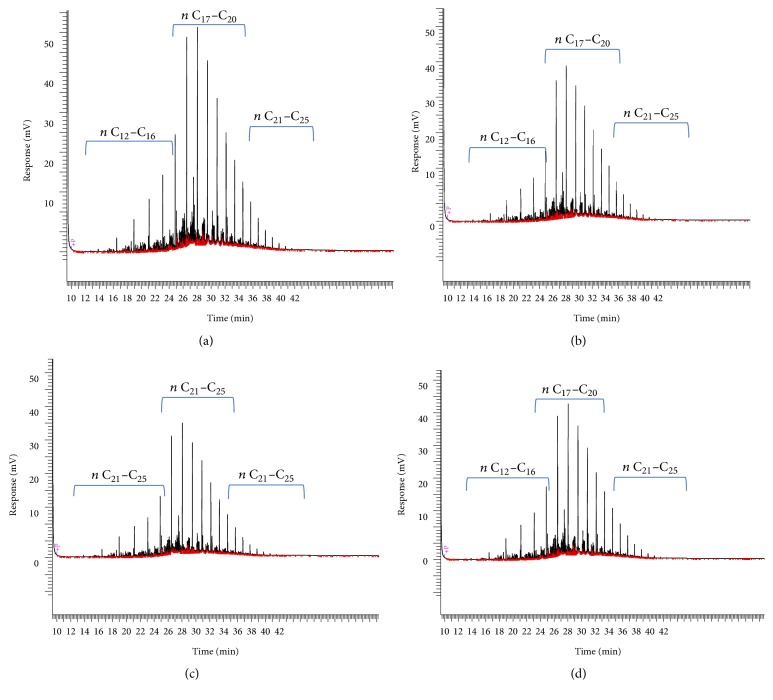
Chromatograms of GC (FID) analysis of control (a), HDB8 (b), HDB9 (c), and HDB18 (d) culture.

The biodegradation of diesel hydrocarbons was monitored by GC analysis of the residual hydrocarbons in the cultural media (MSM) of HDB8 HDB9 and HDB18. The control culture was used as control (abiotic test) as well as to select representative peaks from the chromatograms, covering 3 hydrocarbons ranges: low (*n*-C-12 to* n*- C-16), medium (*n*-C-17 to* n*-C-20), and high (*n*-C-21 to* n*-C-25) molecular weight based on the retention times (Figure 3). The degradation of the corresponding hydrocarbons was expressed as a percentage of area reduction. Figure 3 shows the chromatograms obtained with the 3 isolates HDB8, HDB9, and HDB18 compared to the control culture. Results are shown in Table 4. It is obviously clear that the chromatograms obtained with the 3 isolates were different, showing different removal profiles. The variability shown in the potentialities of the 3 strains to remove diesel hydrocarbons may be a result of variability of their metabolism.

### 3.5. Role of Nitrogen Source Composition and C/N Ratio in the Potentialities of the Isolates to Degrade Diesel Hydrocarbons

In order to study the effect of nutrients composition on the biological activity of the selected strains, three different nitrogen sources, ammonium nitrate (NH_4_NO_3_), ammonium chloride (NH_4_Cl), and sodium nitrate (NaNO_3_), were used at the same concentration of 4 g/L but, due to their nitrogen content, the corresponding C/N ratios were different, of 60/1, 80/1, and 120/1, respectively, in 10% diesel medium, containing 75 g/L total carbons. This may be considered as a comparison between different conditions of growth. Table 4 shows the hydrocarbons removal efficiencies of the 3 ranges of diesel hydrocarbons, obtained by the three isolates at the three cultural conditions. It is clear that the activities were different from one isolate to another and from one condition to another. HDB8 gave similar overall removal efficiency with ammonium nitrate and ammonium chloride but significant differences in the distribution of such efficiencies on separate molecular weight ranges. HDB9 gave totally different efficiencies in the three media, with weak activity in sodium nitrate medium. A similar conclusion may also be drawn for isolate HDB18.

Although the C/N ratios in the three media were not the same, the biological activities and thus metabolisms of the three isolates were showing variability in their potentialities towards 75 g/L carbons. Ammonium nitrate seemed to be the preferable nitrogen source for the three strains.

In order to study the effect of the C/N ratio on the biological activity of the selected strains, ammonium nitrate (NH_4_NO_3_) was used as sole nitrogen source at different concentrations, corresponding to different C/N ratios with fixed total carbon concentration of 75 g/L. Results of hydrocarbons removal by the 3 strains are shown in Table 5.

It is clear that, for each strain, there is a corresponding optimal C/N or a range within which the hydrocarbon removal is optimal.

This growth dependence on the C/N ratio may vary from one nitrogen source to another at 10% diesel. The profile of hydrocarbon degradation of diesel hydrocarbons by each strain at all the conditions is shown in Table 5. These results of variations within the compared strains and conditions were furtherly analyzed for significance using ANOVA (Table 6) and clearly showed that the biological activity towards hydrocarbons varies depending on the nitrogen source and the C/N ratio. Table 6 also shows that the removal of HMW hydrocarbons with strain HDB9 does not change significantly by alternating their nitrogen source, but still highly significantly different when varying C/N Ratios.

However, the results show high variability in the biological activity of the three strains at different conditions. Indeed, with ammonium nitrate, HDB8 was more active on long chain* n*-alkanes at a C/N ratio of 60/1. At low C/N ratio of 40/1, the overall activity was much lower but still high on longer chain* n*-alkanes. With the strain HDB9, by using ammonium nitrate, the highest activity towards all the hydrocarbons was at C/N of 40. The strain HDB18 was not able to efficiently degrade medium chain alkanes, but effective for long chain ones. The highest overall activity was obtained using ammonium nitrate at a C/N ratio of 60/1 for HDB8 and at C/N ratio of 40/1 for HDB9.

## 4. Discussion

It is always expected to follow an isolation and screening program that suits the objective of the application to select microorganisms with expected biological activity. Here, the objective of the isolation program is to build a collection of adapted hydrocarbon-degrading bacteria to weathered oil polluting soils at harsh conditions. It is meaningful that some loss of bacteria in the phase of their purification is expected as a result of losing hydrocarbon-degrading activity, known to be exhibited by large plasmids [31], or because they need cometabolism or they are commensal bacteria [32]. All the isolated ones were supposed to be genetically stable and able to grow individually, to some extent, on oil or diesel components. Three of the isolates are* Pseudomonas aeruginosa*, which are the most effective strains [28, 33]. This result is expected due to the fact that this species is the most involved in bioremediation, particularly of hydrocarbons. There are also several strains of* Arthrobacter*,* Citrobacter*,* Bacillus*, and* Klebsiella*. These species are also reported in the literature for their biological activities involving the bioremediation of hydrocarbon [32, 33]. Unexpectedly, one of our isolates belongs to the* Klebsiella* species. To date, there has been no report on how* Klebsiella*, which is normally pathogenic, exhibits such biological activity. Several strains of* Bacillus cereus* and* Bacillus thuringiensis* also show some activity involving the degradation of hydrocarbons. No studies have reported that* Cronobacter pulveris* or* Cronobacter muytjensii* are involved in the degradation of hydrocarbon.

By diversification of cultural conditions (solid/liquid, oil/diesel) as well as high selection pressure, using 10% diesel in liquid medium allowed selection of hydrocarbon-degrading bacteria exhibiting various biological activities and metabolisms. Some bacteria change their activities from one condition to another, meaning that they are armed with necessary metabolic and enzymatic activities as well as metabolic regulating alternatives. Two strains of* Pseudomonas aeruginosa* isolated from the same soil, polluted with weathered oil, exhibited different metabolic activities. Their adaptation to the harsh condition was through different routes. Indeed, bacteria have been designed to be adaptable. Their surrounding layers and the genetic information for these and other structures associated with a bacterium are capable of many types of adaptation, reflected in the overall metabolism [34]. Some are reversible, disappearing when the particular pressure is lifted. Other alterations are maintained and can even be passed on to succeeding generations of bacteria [34]. However, their respective microenvironments (tarball, adsorption to solids, etc.) might be an additional factor to develop such adaptation route.

The isolates purified through this program tolerate high hydrocarbon concentrations and their potential toxicity. This is expected since the natural environment from where they were isolated was highly polluted with hydrocarbons.

Interestingly, some selected bacteria were shown able to shift their degradative activities from one range molecular weight of hydrocarbons to another, based on the nitrogen source and C/N ration. High molecular hydrocarbons were preferable at conditions specific to each isolate. Differences were observed between 2* Pseudomonas aeruginosa* isolates. This might be expected, since these bacteria were adapted to weathered hydrocarbons, normally with evaporated and degraded low molecular weight hydrocarbons.

On the other hand, considering the chemical instability of weathered hydrocarbons, with modifications in their structures due to oxidation and light occurring in nature, would suggest that the isolated bacteria through our isolation and screening program could represent an appropriate alternative to remediate polluted soils exposed for long time to weathered oil at harsh conditions. By the characterization of the polluting hydrocarbons and their weathering level, the appropriate bacteria would be suggested with the appropriate nitrogen source and C/N ratio. These parameters would vary with the soil characteristics, hydrocarbons composition, and bacterial isolate characterized with nitrogen source and C/N ratio requirements for degradation of the corresponding hydrocarbons.

It was evidently shown from this study that hydrocarbon-degrading bacteria are abundant in the Qatari environment and diversified in weathered hydrocarbon-polluted sites. An appropriate isolation and screening program has been designed to isolate interesting hydrocarbon-degrading bacteria, and in this case, the combination of growth parameters and biological activities resulted in the isolation of 39 bacterial strains. For the isolation and screening, the diversification of media composition, the nitrogen source composition, the C/N ratios, and the application of high pressure selection with high diesel concentrations were useful to direct the isolation of appropriate isolates for a given polluted area. Moreover, the screening program based on isolates' growth capability was combined with the biological activities in removing hydrocarbons from diesel. It was shown that all our isolated strains behave differently.

Further studies were conducted on several selected bacterial strains, which exhibited variations in growth dynamics, biological activities, and tolerance to diesel toxicity. Biological activities shifted from short chain to longer chain* n*-alkanes and vice versa using different nitrogen sources, C/N ratios. These findings have not been reported in prior literature. In an aerobic treatment of nonhazardous organics, the ratio C/N is about 100/5, since about 50% of the carbon are lost by anabolism as CO_2_ and the other 50% are assimilated by anabolism in biomass. With hazardous compounds, it is normally less than that, as stated by Sihag et al. [35]. Moreover, according to Simarro et al. [36], the nutrient ratio required by PAH-degrading bacteria depends on environmental conditions, type of bacteria, and type of hydrocarbons. This is important due to the fact that the conditions of the bioremediation as well as the used bacterium may change according to the composition of hydrocarbons (pollutants); therefore, the pollution parameters should be analyzed to select the strain and the conditions of its implementation. Among our isolates, 3 are* Pseudomonas aeruginosa*. The occurrence of this bacterium in polluted areas is expected, particularly with hydrocarbons. According to our screening program, it appears that these strains are different in terms of their biological activity and growth. Several other species identified within our isolates have also been reported in the literature. There are also several strains of* Arthrobacter* and* Citrobacter* in our collection. These findings clearly show that it is important to consider a specific screening program to select the appropriate isolate to be used in any bioaugmentation strategy for bioremediation of weathered oil in harsh soils at harsh conditions, avoiding frequent failure of bioaugmentation approaches in many regions including the gulf area.

## Figures and Tables

**Table 1:** List and origin of the coded 39 isolated isolates.

Origin of sample	Crude oil-coated MSM plates	Diesel-coated MSM plates
Autoworkshop 1 (lubricants leak)	HDB11, HDB19	HDB7
Autoworkshop 2 (diesel leaking site)		HDB1, HDB10
Autoworkshop 3 (oil waste tanks)	HDB3, HDB8, HDB9, HDB12, HDB15, HDB18, HDB36, HDB37	HDB6
Autoworkshop 4 (repairing area)	HDB2, HDB4, HDB5, HDB13, HDB14, HDB16, HDB17, HDB20	
Oil waste management site 1		HDB26, HDB27, HDB28, HDB32, HDB34
Oil waste management site 2	HDB22, HDB25	HDB31, HDB33
Oil waste management site 3	HDB21, HDB23, HDB24	HDB29, HDB30, HDB35, HDB38, HDB39

**Table 2:** Growth (cfu counts) and removal efficiency of bacterial isolates after 2 weeks of incubation in MSM media supplemented with 10% diesel.

Number	Strain	2 weeks' incubation (×10^7^ cfu/mL)	Removal efficiency (%)
(1)	HDB2	1 ± 1.4	3 ± 0.8
(2)	HDB4	2 ± 0.12	4 ± 0.5
(3)	HDB5	11 ± 0.5	12 ± 2.1
(4)	HDB8	33 ± 1.3	31 ± 3.1
(5)	HDB9	31 ± 1.3	21 ± 2.5
(6)	HDB11	8.2 ± 0.3	10 ± 1.6
(7)	HDB18	20 ± 1.0	10 ± 2.9
(8)	HDB19	1.5 ± 0.06	3 ± 0.3
(9)	HDB38	26 ± 1.0	19 ± 0.7
(10)	HDB39	2 ± 0.1	5 ± 0.1

Values are means of three replicates ±standard deviation.

**Table 3:** Molecular identification of the isolates.

Isolate	Identity	Accession number	Identity
HDB2	*Arthrobacter *sp.	LC093517.1	95%
HDB4	*Citrobacter *sp.	GU451067.1	90%
HDB5	*Citrobacter amalonaticus*	KR063563.1	98%
HDB8	*Pseudomonas aeruginosa*	CP015377.1	98%
HDB9	*Pseudomonas aeruginosa*	JF919950.1	98%
HDB11	*Cronobacter pulveris *	KF534713.1	98%
HDB18	*Citrobacter amalonaticus*	CP014070.1	98%
HDB19	*Cronobacter muytjensii*	CP012268.1	95%
HDB38	*Pseudomonas aeruginosa*	JX962695.1	97%
HDB39	*Klebsiella quasipneumoniae*	CP014696.1	98%

**Table 4:** Hydrocarbon removal efficiency of HDB8, HDB9, and HDB18, with different nitrogen sources, NH_4_NO_3_, NH_4_Cl, and NaNO_3_. Results are averages of 3 culture replicates and 3 separate GC analyses.

Isolate	NH_4_NO_3_	NH_4_Cl	NaNO_3_
*HDB8*			
Total hydrocarbons removal (%)	30 ± 3.1	31 + 2.4	6 ± 0.3
LMW hydrocarbons (%)	39 ± 4.9	28 ± 3.7	0 ± 1.2
MMW hydrocarbons (%)	29 ± 3.9	33 ± 2.6	3 ± 1.7
HMW hydrocarbons (%)	24 ± 4.6	29 ± 4.0	14 ± 2.4
*HDB9*			
Total hydrocarbons removal ( %)	21 ± 2.5	8 ± 1.3	3 ± 1.0
LMW hydrocarbons (%)	35 ± 4.1	11 ± 2.6	0 ± 0.5
MMW hydrocarbons (%)	21 ± 3.9	5 ± 1.7	0 ± 0.5
HMW hydrocarbons (%)	6 ± 2.9	9 ± 1.2	9 ± 1.6
*HDB18*			
Total hydrocarbons removal (%)	10 ± 2.9	19 ± 1.6	3 ± 0.8
LMW hydrocarbons (%)	16 ± 3.0	33 ± 1.7	8 ± 2.2
MMW hydrocarbons (%)	14 ± 1	22 ± 2.5	0 ± 0.5
HMW hydrocarbons (%)	4 ± 0.6	2 ± 1.2	0 ± 0.2

LMW: low molecular weight; MMW: medium molecular weight; HMW: high molecular weight.

**Table 5:** Hydrocarbon removal efficiency of HDB8, HDB9, and HDB18 with NH_4_NO_3_ at different C : N ratios: 70 : 1, 60 : 1, 50 : 1, and 40 : 1. Results are averages of 3 culture replicates and 3 separate GC analyses.

C/N	C/N 70/1	C/N 60/1	C/N 50/1	C/N 40/1
*HDB8*				
Total hydrocarbons removal (%)	11 ± 2.6	30 ± 1.4	16 ± 2.5	20 ± 3.4
LMW hydrocarbons (%)	20 ± 3.4	39 ± 4.5	15 ± 2.2	20 ± 3.3
MMW hydrocarbons (%)	12 ± 3.1	29 ± 2.9	19 ± 3.7	20 ± 2.9
HMW hydrocarbons (%)	7 ± 2.2	24 ± 4.1	15 ± 2.2	20 ± 1.9
*HDB9*				
Total hydrocarbons removal (%)	10 ± 1.6	21 ± 0.7	10 ± 1.2	38 ± 2.9
LMW hydrocarbons (%)	2 ± 0.9	35 ± 4.1	0 ± 0.5	45 ± 3.3
MMW hydrocarbons (%)	21 ± 2.5	21 ± 3.7	28 ± 3.4	37 ± 4.2
HMW hydrocarbons (%)	6 ± 1.7	6 ± 2.5	2 ± 1.2	32 ± 2.2
*HDB18*				
Total hydrocarbons removal (%)	11 ± 2.1	10 ± 2.9	8 ± 1.7	13 ± 2.1
LMW hydrocarbons (%)	0 ± 0.5	16 ± 3.0	4 ± 0.8	23 ± 1.7
MMW hydrocarbons (%)	29 ± 3.1	14 ± 1	1 ± 0.5	16 ± 1.7
HMW hydrocarbons (%)	6 ± 2.1	4 ± 0.6	19 ± 2.1	7 ± 1.7

LMW: low molecular weight; MMW: medium molecular weight; HMW: high molecular weight.

**Table 6:** Statistical analysis of the significance of hydrocarbon removal efficiency of HDB8, HDB9, and HDB18, with different nitrogen sources and C/N ratios.

	Nitrogen sources	*P* value at 95% confidence	C : N ratios	*P* value at 95% confidence
*HDB8*				
Total removal (average)	*∗∗∗*	4.42*E* − 06	*∗∗*	4.25*E* − 04
LMW hydrocarbons	*∗∗∗*	6.40*E* − 05	*∗∗*	0.000596589
MMW hydrocarbons	*∗∗∗*	1.76*E* − 05	*∗∗*	0.004427376
HMW hydrocarbons	*∗*	0.01	*∗∗*	0.001089686
*HDB9*				
Total removal (average)	*∗∗∗*	6.56*E* − 06	*∗∗∗*	9.85*E* − 07
LMW hydrocarbons	*∗∗∗*	3.86*E* − 06	*∗∗∗*	2.72*E* − 07
MMW hydrocarbons	*∗∗∗*	4.73*E* − 05	*∗∗*	0.005285635
HMW hydrocarbons	NS	0.08	*∗∗∗*	1.2505*E* − 06
*HDB18*				
Total removal (average)	*∗∗∗*	5.80*E* − 05	*∗*	0.021019569
LMW hydrocarbons	*∗∗∗*	2.80*E* − 05	*∗∗∗*	1.56095*E* − 06
MMW hydrocarbons	*∗∗∗*	3.64*E* − 05	*∗∗∗*	2.7128*E* − 06
HMW hydrocarbons	*∗*	0.04	*∗∗*	0.000160478

NS: not significant. ^*∗*^Significant. ^*∗∗*^Highly significant. ^*∗∗∗*^Very highly significant.

LMW: low molecular weight; MMW: medium molecular weight; HMW: high molecular weight.
